# circRNA-Associated ceRNA Regulatory Networks in Cardiac Responses to High-Altitude Hypoxia in Tibetan Pigs (*Sus scrofa*)

**DOI:** 10.3390/ijms27104392

**Published:** 2026-05-14

**Authors:** Pan Li, Wei Cheng, Peng Shang, Zhu Tao, Hao Zhang, Bo Zhang

**Affiliations:** 1 Frontiers Science Center for Molecular Design Breeding (MOE), China Agricultural University, Beijing 100193, China; lipan1011@cau.edu.cn (P.L.); chengwei@cau.edu.cn (W.C.); taozhu2020@cau.edu.cn (Z.T.); hzhang@cau.edu.cn (H.Z.); 2 National Engineering Laboratory for Animal Breeding, College of Animal Science and Technology, China Agricultural University, Beijing 100193, China; 3 Beijing Key Laboratory for Animal Genetic Improvement, College of Animal Science and Technology, China Agricultural University, Beijing 100193, China; 4 Department of Animal Husbandry, Xizang Agricultural and Animal Husbandry University, Linzhi 860000, China; shangpeng1984@xza.edu.cn

**Keywords:** Tibetan pigs, hypoxic adaptation, circRNAs, ceRNA network, HIF-1 signaling

## Abstract

High-altitude hypoxic adaptation in mammals involves complex molecular mechanisms, with non-coding RNAs (ncRNAs) increasingly reported to participate in hypoxia-related regulation. However, the contribution of circRNAs in cardiac adaptation to chronic hypoxia remains largely unexplored. This study performed an integrative competitive endogenous RNA (ceRNA) analysis to investigate circRNA-mediated regulatory networks in the hearts of Tibetan pigs and Yorkshire pigs maintained under high- and low-altitude conditions, using four comparison groups (TH, TL, YH, and YL). Using Ribo-Zero RNA sequencing, we identified 961 circRNAs in heart tissues, with 358 differentially expressed circRNAs (DE-circRNAs) detected across the four groups. Functional enrichment analysis revealed that their host genes were associated with hypoxia-related pathways, including HIF-1, VEGF, AMPK, and autophagy, critical for energy metabolism and mitochondrial function. A HIF-1-specific ceRNA network was constructed, identifying key axes including circDUSP16–ssc-miR-671-5p–*CAMK2A*, circTLK1–ssc-miR-331-3p–*SERPINE1*, and circTLK1–novel-miR-624–*ENO1*. JASPAR analysis predicted potential HIF-1α binding sites in the promoters of *ENO1*, *SERPINE1*, and *CAMK2A*, supporting their regulatory roles. These findings provide a transcriptomic overview of circRNA expression patterns in pig heart tissues under different altitude conditions and prioritize candidate ceRNA relationships for further functional investigation.

## 1. Introduction

High-altitude environments, generally referring to regions above approximately 2500 m above sea level, are characterized by reduced barometric pressure, lower ambient temperatures, and limited oxygen availability [1,2]. Importantly, the fraction of oxygen in the atmosphere remains nearly constant at approximately 20.9%; however, the decline in barometric pressure at high altitude reduces the partial pressure of inspired oxygen, resulting in hypobaric hypoxia. For example, compared with near-sea-level lowland environments, where inspired oxygen partial pressure is approximately 21 kPa, this value decreases to approximately 14–15 kPa at around 3000 m above sea level. Such hypoxic conditions impose substantial physiological challenges on mammals adapted to lowland environments, disrupting metabolic homeostasis and requiring adaptive mechanisms to maintain oxygen transport, energy metabolism, and cardiovascular function [3,4]. Research leveraging the unique geography of high-altitude regions, such as the Qinghai–Tibet Plateau, has significantly advanced our understanding of hypoxia’s impact on mammalian physiology, offering valuable insights into biological adaptations and potential medical applications for hypoxia-related disorders, including cardiovascular diseases and altitude sickness [5,6]. The Tibetan pig (*Sus scrofa*) is a domestic indigenous pig breed primarily distributed at altitudes above 2500 m on the Qinghai–Tibet Plateau and has been reported to exhibit physiological characteristics associated with long-term residence in high-altitude environments, showing differences from lowland pig breeds in traits related to hypoxia-associated physiological responses [7,8,9]. These characteristics include differences in cardiovascular, respiratory, and hematopoietic functions, such as arterial structure and blood biochemical parameters, which are associated with oxygen transport and utilization [10,11,12]. Owing to these features, the Tibetan pig serves as a useful domestic animal model for investigating molecular responses to hypoxic conditions.

Despite advancements in understanding high-altitude adaptations, significant challenges persist in unraveling the role of non-coding RNAs (ncRNAs) in this process. While studies have identified DNA methylation, proteomic, mRNA, and miRNA expression profiles in Tibetan pig hearts, the comprehensive interplay between coding and non-coding RNAs, particularly circRNAs, remains elusive [8,13,14]. The advent of high-throughput sequencing has transformed our perception of ncRNAs, once dismissed as non-functional, revealing their pivotal roles in regulating cellular physiology and pathology [15,16]. Among ncRNAs, circRNAs stand out due to their closed-loop structure, which confers greater stability than linear RNAs like lncRNAs and miRNAs, and their ability to act as miRNA sponges within competitive endogenous RNA (ceRNA) networks [17]. While lncRNAs and miRNAs have been implicated in hypoxia responses, metabolic regulation, and disease progression in various species, their roles in Tibetan pigs are underexplored [18,19,20]. For instance, studies on yaks and Tibetan chickens have identified lncRNAs involved in hypoxic adaptation, suggesting their potential in regulating oxygen transport and signaling pathways [21,22,23]. However, the specific contributions of circRNAs in the heart, a key organ governing oxygen delivery and hypoxic responses, remain poorly understood in Tibetan pigs. Elucidating these mechanisms is essential for understanding the integrated molecular regulation of hypoxic adaptation.

This study aims to explore the role of circRNAs in the hypoxic adaptation of Tibetan pig hearts by examining their expression profiles and regulatory functions within a ceRNA network. We constructed a detailed ceRNA network in Tibetan pig heart tissues, utilizing previously identified differentially expressed miRNAs (DE-miRNAs) and mRNAs (DE-mRNAs) from our earlier studies [24], to pinpoint key regulatory axes that influence energy metabolism, vascular stability, and calcium signaling under hypoxic stress. Given the heart’s critical role in circulatory adaptation, it provides an optimal focus for elucidating how Tibetan pigs achieve enhanced hypoxic tolerance compared to lowland breeds, as demonstrated by their superior cardiac function [8]. Our findings are expected to offer new perspectives on the molecular mechanisms driving hypoxic adaptation, deepening our understanding of cardiac resilience in high-altitude settings and potentially guiding therapeutic approaches for hypoxia-related cardiovascular conditions.

## 2. Results

### 2.1. Identification and Characterization of circRNAs in Pig Heart Tissue

**Figure 1:**
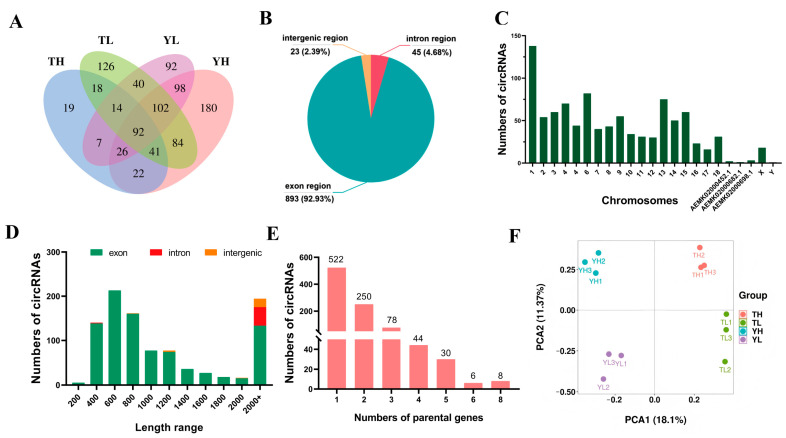
Identification and characterization of circRNAs in heart tissues of pigs. (**A**) Venn diagram of circRNAs in four groups of pig heart tissues; (**B**) Distribution of genomic regions from which circRNAs have been identified; (**C**) Distribution of the identified circRNAs in different pig chromosomes; (**D**) Length distribution of the identified circRNAs; (**E**) The numbers of circRNAs derived from parental gene; (**F**) The PCA plot of 12 samples using all circRNA expression.

We profiled the circRNA landscape in heart tissues from TH, YH, TL, and YL by characterizing circRNA transcripts from 12 rRNA-depleted RNA-Seq samples. Sequencing data have been deposited in the Sequence Read Archive (SRA) under the accession number PRJNA880668. After removing and filtering adapters, poly-N, and low-quality reads, a total of 1,347,697,948 clean reads were obtained. These clean reads yielded numerous back-spliced junction reads across samples (Table S1). Across individual samples, approximately 774–3052 circRNA candidates were detected using find_circ and CIRI2 (Table S2). After merging circRNAs across the 12 samples and applying filtering criteria, 961 non-redundant high-confidence circRNAs were retained for downstream analysis. These circRNAs were derived from 692 parental genes, including 35 novel genes and 657 annotated genes. At the group level, 239, 645, 517, and 471 circRNAs were detected in TH, YH, TL, and YL, respectively. Among them, 19, 180, 126, and 92 circRNAs were specifically expressed in TH, YH, TL and YL, respectively (Figure 1A, Table S3). The retained circRNAs were predominantly exonic (893, 93%), followed by intronic (45, 5%) and intergenic (23, 2%) types, suggesting that most circRNAs are derived from protein-coding exons (Figure 1B). CircRNA lengths ranged from 70 nt to 99,375 nt, with the majority mapping to chromosome 1 (Figure 1C,D). Among circRNA–parental gene pairs, 522 circRNAs arose from a unique parental gene, underscoring the preference of parental genes to produce a single circRNA (Figure 1E). Principal component analysis (PCA) was performed using all circRNA expressions from 12 samples, and the result showed clear classification among different groups, which indicated the high quality and reproducibility of our datasets (Figure 1F).

### 2.2. Expression Profiles of circRNAs Across Different Groups

**Figure 2:**
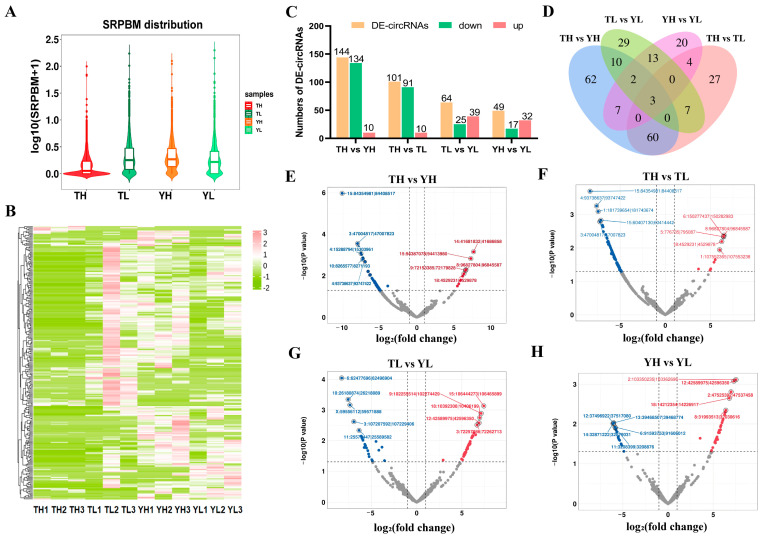
Description of DE-circRNAs in four comparison groups (TH vs. YH, TH vs. TL, TL vs. YL, and YH vs. YL). (**A**) Distribution of circRNA expression values of four groups. The ordinate shows the log_10_(SRPBM + 1) of each RNA from the four groups of RNA-seq data; the middle line in the box represents the median of SRPBM. (**B**) Heatmap of DE-circRNAs in four comparisons. Different columns in the Figure represent different samples, and different rows represent different circRNAs. The color represents the expression level of circRNA in samples Log_10_ (SRPBM+0.000001). (**C**) Histogram of four comparisons of the number of DE-circRNAs. (**D**) Venn diagram of DE-circRNAs among the four comparison groups. (**E**) Volcano map of DE-circRNAs in TH vs. YH. (**F**) Volcano map of DE-circRNAs in TH vs. TL. (**G**) Volcano map of DE-circRNAs in TL vs. YL. (**H**) Volcano map of DE-circRNAs in YH vs. YL. DE-circRNAs were defined using the criteria of |log_2_(fold change)| ≥ 1 and adjusted *p* < 0.05. Significantly upregulated and downregulated DE-circRNAs are marked in red and blue, respectively.

We normalized the circRNA expression profiles using spliced reads per billion mapped reads (SRPBM) values to enable quantitative comparisons across samples. As shown in Figure 2A, the TH group showed a lower overall circRNA expression distribution than the YH, TL, and YL groups. The DE-circRNAs were visualized using a heatmap, showing distinct expression patterns across the four comparison groups, with samples clustered by group and circRNAs ordered by expression levels (Figure 2B). Using the predefined criteria of |log_2_ (fold change)| ≥ 1 and adjusted *p* < 0.05, we identified 358 statistically significant DE-circRNAs across the four pairwise comparisons (Figure 2C, Table S4). Specifically, 144 DE-circRNAs were detected in TH vs. YH, 101 in TH vs. TL, 64 in TL vs. YL, and 49 in YH vs. YL. Among them, 134, 91, 25, and 17 circRNAs were downregulated, while 10, 10, 39, and 32 circRNAs were upregulated in these four comparisons, respectively (Figure 2C). Venn diagram analysis showed that 63 DE-circRNAs were shared between TH vs. YH and TH vs. TL comparisons. In addition, 15 DE-circRNAs were shared between TH vs. YH and TL vs. YL, whereas 7 DE-circRNAs overlapped between YH vs. YL and TH vs. TL (Figure 2D). Volcano plots further illustrated the distribution of significantly upregulated and downregulated DE-circRNAs in each pairwise comparison: TH vs. YH (Figure 2E), TH vs. TL (Figure 2F), TL vs. YL (Figure 2G), and YH vs. YL (Figure 2H). Based on these statistically significant DE-circRNAs, TH-associated DE-circRNAs were selected for subsequent functional enrichment and ceRNA network analyses.

### 2.3. Experimental Validation of Identified circRNAs

**Figure 3:**
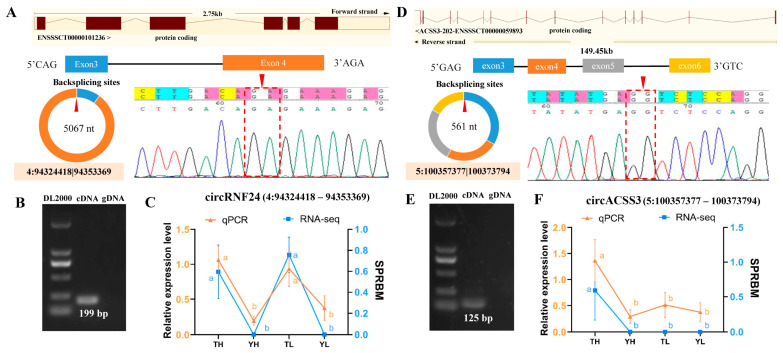
Experimental validation of two randomly selected circRNAs. (**A**) Schematic of 4:94324418|94353369 (*RNF24* gene, exons 3–4, 5067 nt) with Sanger sequencing confirming the back-splice junction (BSJ). The red dashed box highlights the BSJ where the 3’ end of exon 4 joins the 5’ end of exon 3. (**B**) Agarose gel electrophoresis showing PCR products (199 bp) of circRNF24 in cDNA but not gDNA. (**C**) Expression of circRNF24 in heart tissues of TH, YH, TL, and YL by RT-qPCR (primary Y-axis) and RNA-seq (secondary Y-axis). Data are shown as mean ± SE; different letters (a, b) indicate *p* < 0.01. (**D**) Schematic of 5:100357377|100373794 (*ACSS3* gene, exons 3–6, 561 nt) with Sanger sequencing confirming the BSJ. The red dashed box highlights the BSJ where the 3’ end of exon 6 joins the 5’ end of exon 3. (**E**) Agarose gel electrophoresis showing PCR products (125 bp) of circACSS3 in cDNA but not gDNA. (**F**) Expression of circACSS3 in heart tissues of TH, YH, TL, and YL by RT-qPCR (primary Y-axis) and RNA-seq (secondary Y-axis). Data are shown as mean ± SEM from three biological replicates; different letters (a, b) indicate *p* < 0.01. TH: Tibetan pig at high altitude; TL: Tibetan pig at low altitude; YH: Yorkshire pig at high altitude; YL: Yorkshire pig at low altitude.

To validate the reliability of our circRNA identification and differential expression analysis, two representative circRNAs were selected for experimental validation: 4:94324418–94353369 (5067 nt, derived from exons 3–4 of the *RNF24* gene) and 5:100357377–100373794 (561 nt, spanning exons 3–6 of the *ACSS3* gene). Sanger sequencing confirmed the back-splice junctions of both circRNAs (Figure 3A,B). Agarose gel electrophoresis showed specific PCR products of 199 bp and 125 bp from cDNA templates but no amplification from genomic DNA (gDNA), supporting their circular structure (Figure 3B,E). Additionally, qRT-PCR was performed to verify the expression patterns of these circRNAs in heart tissues from TH, YH, TL, and YL groups. The qRT-PCR results showed expression trends consistent with the RNA-seq data, with significant differences across the groups (*p* < 0.01) (Figure 3C,F). Together, these results support the reliability of the circRNA detection pipeline and the reproducibility of the differential expression profiles identified in this study.

### 2.4. Functional Enrichment Analysis of DE-circRNA Host Genes

**Figure 4:**
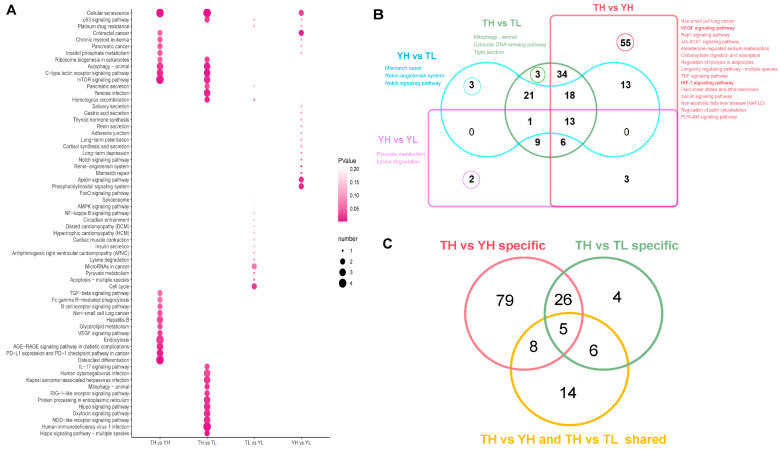
KEGG Pathway Enrichment Analysis of DE-circRNA Host Genes. (**A**) Bubble plot of KEGG pathways enriched across the four comparison groups (TH vs. YH, TH vs. TL, TL vs. YL, YH vs. YL). The x-axis represents the comparison groups, the y-axis lists the enriched pathways, bubble size indicates the number of genes, and color represents the *p*-value. (**B**) Venn diagram of overlapping KEGG pathways among the four comparison groups. Representative significantly enriched pathways are listed for each group. (**C**) Venn diagram of TH-specific DE-circRNA host gene pathways.

To describe the pathway distribution of the 358 identified DE-circRNAs, we performed KEGG pathway enrichment analysis on their corresponding host genes across four comparison groups (TH vs. YH, TH vs. TL, TL vs. YL, YH vs. YL) (Table S5). In the TH vs. YH group, 144 DE-circRNAs (e.g., circDUSP16, circTLK1, circPIK3CB, circNFATC2, circTGFBR1) were primarily enriched in hypoxia-responsive pathways, including VEGF signaling, glycolysis/gluconeogenesis, and autophagy (animal), which are central to angiogenesis, energy metabolism, and cellular stress responses. For TH vs. TL, 101 DE-circRNAs were associated with the AMPK signaling pathway and mitophagy (animal), implying critical roles in maintaining energy homeostasis and mitochondrial quality control in hypoxic cardiac tissue. In contrast, TL vs. YL showed enrichment in pyruvate metabolism and cardiac muscle contraction, while YH vs. YL was enriched in the renin-angiotensin system and adherens junctions, reflecting general physiological regulatory processes (Figure 4A). Cross-comparison analysis identified 13 conserved pathways (e.g., cellular senescence, p53 signaling pathway) across all four groups. Notably, the HIF-1 signaling and insulin signaling pathways were uniquely enriched in TH vs. YH, whereas the tight junction pathway was specific to TH vs. TL (Figure 4B). Focusing on TH-specific DE-circRNAs, host genes of the 62 unique DE-circRNAs in TH vs. YH were enriched in 118 pathways (79 unique), including VEGF signaling, AMPK signaling, and mTOR signaling. The 27 unique DE-circRNAs in TH vs. TL were enriched in 41 pathways (4 unique), dominated by mitophagy (animal). Additionally, the 60 DE-circRNAs shared between TH vs. YH and TH vs. TL were enriched in 33 pathways (14 unique), such as autophagy (animal) and glycerolipid metabolism (Figure 4C, Table S6). Collectively, these results showed that the host genes of TH-specific DE-circRNAs were significantly enriched in pathways related to angiogenesis, mitochondrial quality control, and energy metabolism. Based on these enrichment profiles, 122 TH-associated DE-circRNAs (62 unique + 60 shared) were selected for subsequent ceRNA network construction.

### 2.5. Construction of a Hypoxia-Related ceRNA Regulatory Network

**Figure 5:**
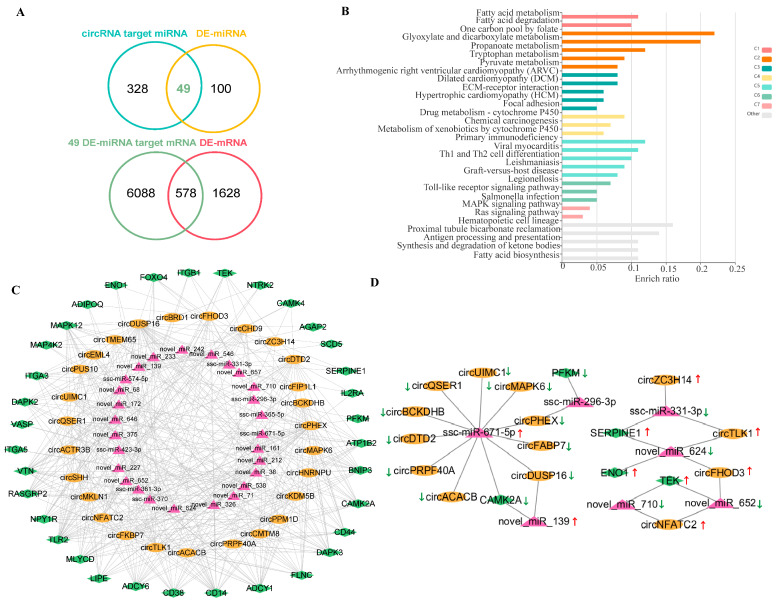
ceRNA Network and Functional Analysis of TH-Specific DE-circRNAs. (**A**) Network plot of the initial ceRNA network (36 DE-circRNAs, 44 DE-miRNAs, 578 DE-mRNAs). (**B**) KEGG enrichment bar plot of the 578 DE-mRNAs. Bars indicate the enrichment ratio of each pathway. Different colors represent pathway clusters (C1–C7), whereas gray bars indicate pathways classified as “Other”. Representative enriched pathways were associated with fatty acid metabolism, carbon metabolism, amino acid metabolism, cardiac function, immune response, and signaling pathways. (**C**) Hypoxia-related ceRNA network with 27 DE-circRNAs, 27 DE-miRNAs, and 34 DE-mRNAs. (**D**) HIF-1 signaling–specific ceRNA subnetwork including 14 DE-circRNAs, 7 DE-miRNAs, and 5 DE-mRNAs. Yellow, red, and green nodes represent circRNAs, miRNAs, and mRNAs, respectively. Red upward arrows denote upregulation, and green downward arrows denote downregulation in the TH group.

To construct a candidate ceRNA network, we first selected 122 TH-associated DE-circRNAs (62 unique in TH vs. YH and 60 shared between TH vs. YH and TH vs. TL) based on differential expression patterns and the KEGG enrichment profiles of their host genes in hypoxia-related pathways. Using miRanda and TargetScan, we predicted 377 miRNAs potentially targeted by these circRNAs. By intersecting these with DE-miRNAs from our previous TH vs. YH study, we identified 49 DE-miRNAs, forming 188 circRNA–miRNA pairs. Subsequent target prediction for these DE-miRNAs generated 6666 mRNAs (21,361 DE-miRNA–mRNA pairs), which were filtered against TH vs. YH DE-mRNAs to yield 578 DE-mRNAs and 1977 DE-miRNA–DE-mRNA pairs. The initial ceRNA network comprised 36 DE-circRNAs, 44 DE-miRNAs, and 578 DE-mRNAs, with 156 circRNA-miRNA pairs and 1978 miRNA–mRNA pairs (Figure 5A). Notably, 13 DE-circRNAs interacted with more than three miRNAs, with circDUSP16 targeting a maximum of 24 miRNAs (Table S7). Next, KEGG enrichment analysis of the 578 DE-mRNAs identified pathways associated with hypoxia-related biological processes, including metabolic reprogramming (Fatty acid metabolism, Propanoate metabolism), cardiac remodeling (ARVC, DCM, HCM, ECM-receptor interaction), and inflammation (Th1/Th2 cell differentiation, Toll-like receptor signaling) (Figure 5B, Table S8). Based on KEGG results and literature, we selected 152 DE-mRNAs associated with hypoxia-related pathways to construct a candidate hypoxia-related ceRNA network, comprising 27 DE-circRNAs, 27 DE-miRNAs, and 34 DE-mRNAs, with 87 circRNA–miRNA pairs and 103 miRNA–mRNA pairs (Figure 5C). To specifically investigate the HIF-1 signaling pathway, we extracted five DE-mRNAs (*ENO1*, *PFKM*, *TEK*, *SERPINE1*, *CAMK2A*) and built a HIF-1-specific subnetwork. This subnetwork included 14 DE-circRNAs, 7 DE-miRNAs, and the 5 DE-mRNAs, forming 20 circRNA–miRNA pairs and 9 miRNA–mRNA pairs (Figure 5D, Table S9). Specifically, *ENO1* was regulated by novel_miR_624 via circTLK1 and circFHOD3; *PFKM* by ssc-miR-296-3p via circPHEX; *TEK* by novel-miR-652 through circNFATC2 and circFHOD3, and by novel-miR-710 via circNFATC2; *SERPINE1* by ssc-miR-331-3p through circZC3H14, and by novel_miR_624 through circTLK1; and *CAMK2A* by ssc-miR-671-5p via 10 circRNAs (e.g., circDUSP16, circPHEX) and by novel-miR-139 through circDUSP16.

### 2.6. Functional Analysis Specific ceRNA Subnetwork in HIF-1 Signaling Pathway

**Figure 6:**
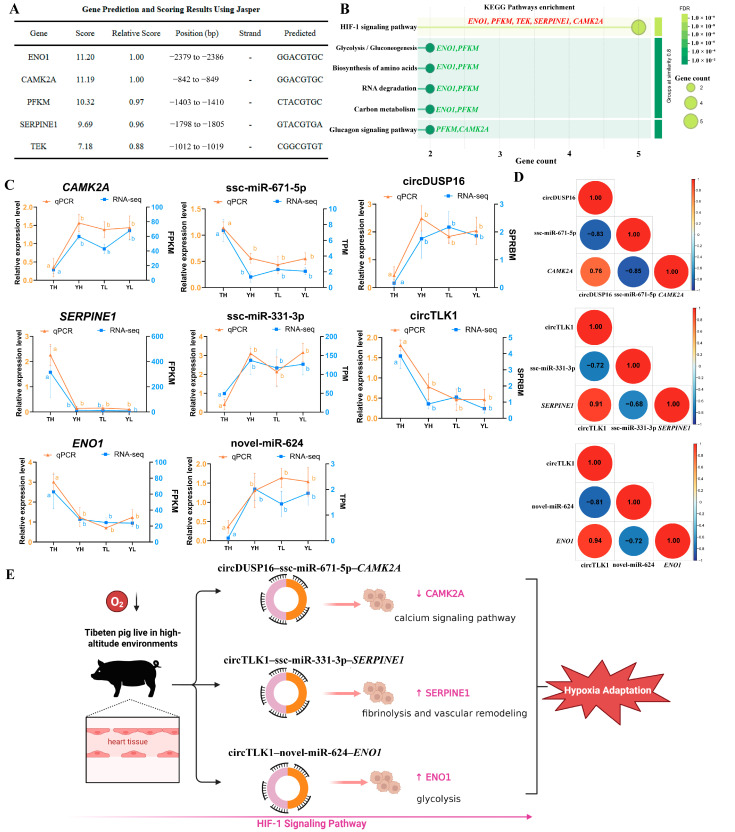
Functional Analysis and Proposed Model of Hypoxic Adaptation in Tibetan Pig Heart. (**A**) Predicted HIF-1α binding motifs in promoter regions of *ENO1*, *SERPINE1*, *TEK*, *PFKM*, and *CAMK2A* using JASPAR. (**B**) KEGG enrichment analysis of the five DE-mRNAs. (**C**) qPCR validation of the three core ceRNA regulatory axes in Tibetan pig heart, verifying the expression of key molecules (circDUSP16, circTLK1, ssc-miR-671-5p, ssc-miR-331-3p, novel-miR-624, *CAMK2A*, *ENO1*, *SERPINE1*) and confirming their consistent trends with RNA-seq results (a, b indicates significant differences, *p* < 0.05). (**D**) Pearson correlation analysis of the internal components within the three prioritized ceRNA regulatory axes. Heatmaps display the Pearson correlation coefficients (r) calculated across four groups (TH, YH, TL, and YL). The color scale indicates the strength and direction of the correlations, where red denotes a positive correlation and blue denotes a negative correlation. (**E**) Proposed model of hypoxic adaptation in Tibetan pig heart, illustrating the ceRNA regulatory axes and their roles in energy metabolism (*ENO1*), angiogenesis and vascular remodeling (*SERPINE1*), and calcium signaling (*CAMK2A*). The upward arrows denote upregulation, and the downward arrows denote downregulation of the respective genes in Tibetan pigs under hypoxic conditions. Created in BioRender. Li, P. (2026) https://BioRender.com/75vnj8u.

To further examine the candidate HIF-1-related ceRNA subnetwork, we analyzed five DE-mRNAs (*ENO1*, *PFKM*, *TEK*, *SERPINE1*, *CAMK2A*) within the HIF-1 signaling pathway. Using the JASPAR database, we predicted potential HIF-1α binding sites in their promoter regions, revealing high-confidence motifs with scores ranging from 7.18 (*TEK*) to 11.20 (*ENO1*) and relative scores of 0.88–1.00 (Figure 6A). *ENO1* and *CAMK2A* shared the highest-scoring motif (GGACGTGC), suggesting potential HIF-1α binding, while TEK’s distinct motif (CGGCGTGT) suggests nuanced regulation. Functional enrichment analysis showed that these genes were associated with the HIF-1 signaling pathway, suggesting their potential relevance to hypoxia-related responses. *ENO1* and *PFKM* were also enriched in glycolysis/gluconeogenesis, carbon metabolism, biosynthesis of amino acids, and RNA degradation, indicating their involvement in energy metabolism and RNA turnover under hypoxia. *PFKM* and *CAMK2A* were associated with the glucagon signaling pathway, linking energy metabolism to calcium signaling in cardiac tissue. *SERPINE1* and *TEK* were enriched in pathways related to fibrinolysis and vascular remodeling (Figure 6B). Based on the predicted miRNA-binding relationships of circDUSP16 (13 targeted miRNAs) and the significant expression changes in downstream functional mRNAs in TH vs. YH, we prioritized three core ceRNA regulatory axes (circDUSP16–ssc-miR-671-5p–*CAMK2A*, circTLK1–ssc-miR-331-3p–*SERPINE1*, and circTLK1–novel-miR-624–*ENO1*) for experimental validation. To experimentally validate these axes, qPCR was performed on core components across the four groups, with RNA-seq data as reference. Results showed consistent expression trends between qRT-PCR and RNA-seq, and the reciprocal expression patterns were consistent with the predicted ceRNA relationships (Figure 6C). For circDUSP16–ssc-miR-671-5p–*CAMK2A*, downregulated circDUSP16 in TH positively correlated with reduced *CAMK2A* (log_2_FC = −1.90, *p* < 0.01), while ssc-miR-671-5p was upregulated, consistent with negative regulation of both. In circTLK1–ssc-miR-331-3p–*SERPINE1*, upregulated circTLK1 in TH positively correlated with elevated *SERPINE1* (log_2_FC = 3.89, *p* < 0.01), while ssc-miR-331-3p was downregulated, consistent with the predicted circRNA–miRNA–mRNA relationship involving *SERPINE1*. For circTLK1–novel-miR-624–*ENO1*, upregulated circTLK1 in TH positively correlated with increased *ENO1* (log_2_FC = 1.24, *p* < 0.01), while novel-miR-624 was downregulated, consistent with the predicted circRNA–miRNA–mRNA relationship involving *ENO1*. To further evaluate the expression correlations among components of these predicted interactions, Pearson correlation analysis was performed on the internal components of each axis across four groups (Figure 6D). Consistent with the ceRNA sponge hypothesis, robust negative correlations were observed between circRNAs and their targeted miRNAs, while circRNAs and their corresponding mRNAs exhibited strong positive synergies. Specifically, the correlation coefficient between circTLK1 and *ENO1* reached 0.94, while circTLK1 showed a significant negative correlation with novel-miR-624 (r = −0.81). Regarding the vascular remodeling axis, circTLK1 was strongly positively correlated with *SERPINE1* (r = 0.91), with its intermediate ssc-miR-331-3p exhibiting the expected antagonism. Furthermore, the calcium signaling axis was substantiated by the negative correlation between circDUSP16 and ssc-miR-671-5p (r = −0.83) and the positive synergy between circDUSP16 and *CAMK2A* (r = 0.76). These correlation patterns supported the prioritization of these three axes as candidate ceRNA regulatory relationships. Collectively, the qRT-PCR results were consistent with the RNA-seq findings and provided expression-level support for the predicted circRNA–miRNA–mRNA axes. These candidate axes were associated with genes involved in energy metabolism (*ENO1*), fibrinolysis/vascular remodeling (*SERPINE1*), and calcium signaling (*CAMK2A*). This analysis prioritized candidate coding and non-coding RNA interactions for further functional investigation.

## 3. Discussion

Hypoxic adaptation is a crucial mechanism for the survival of high-altitude species, but the role of non-coding RNAs, particularly circRNAs, remains largely unexplored in this process. Although previous studies have primarily focused on coding RNAs in high-altitude mammals such as yaks [25], Tibetan pig [8] and Tibetan sheep [26], this study addresses a significant gap by investigating the regulatory roles of circRNAs in the hypoxic adaptation of Tibetan pig hearts. The pronounced attenuation of the circRNA profile observed in the TH cohort corroborates established paradigms of adaptive non-coding RNA repression under chronic hypoxia, as evidenced in high-altitude ruminants. This suggests that circRNAs may contribute to energy conservation under chronic hypoxia [27]. Unlike studies that focused primarily on coding genes, our results suggest that circRNAs may be involved in hypoxia-related cardiac responses through candidate ceRNA regulatory relationships, providing new insights into cardiac protection through non-coding RNA-mediated regulation in high-altitude environments [28,29].

This study provides an initial characterization of circRNA expression profiles in Tibetan pig heart tissues under high-altitude hypoxic conditions. Among the 961 identified circRNAs, the majority were exonic, consistent with their prevalence in eukaryotic tissues due to biogenesis via back-splicing of pre-mRNA [30,31]. The dominance of exonic circRNAs indicates their stability and regulatory capacity in the cytoplasm, where they can interact with miRNAs to form ceRNA networks [32]. The observed downregulation in the TH group may reflect selective suppression of circRNA biogenesis under chronic hypoxia, possibly mediated by hypoxia-induced changes in splicing factors or RNA-binding proteins, warranting further research. Functional enrichment analysis of DE-circRNA showed host genes revealed significant enrichment in pathways such as VEGF signaling, AMPK signaling, and autophagy, which are critical for angiogenesis, energy homeostasis, and mitochondrial quality control under hypoxic conditions. These pathways align with those identified in yaks, where HIF-1 signaling supports vascular remodeling and metabolic adjustments in heart tissue under high-altitude conditions [22]. Recent transcriptomic and proteomic analyses in Tibetan pigs further corroborate these findings, showing that HIF-1 signaling, glycolysis, and mitochondrial function-related pathways are significantly enriched in lung tissues, highlighting the systemic role of metabolic adaptation in hypoxic conditions [33]. Our study extends these observations by demonstrating that circRNAs play a pivotal role in modulating these pathways through their host genes, emphasizing their regulatory significance in hypoxic adaptation.

The candidate HIF-1-related ceRNA subnetwork, comprising 14 DE-circRNAs, 7 DE-miRNAs, and 5 DE-mRNAs (*ENO1, PFKM, TEK, SERPINE1, CAMK2A*), suggests potential regulatory interactions [34,35]. JASPAR analysis predicted potential HIF-1α binding sites in the promoter regions of these mRNAs, with high-confidence motifs (scores: 7.18 to 11.20; relative scores: 0.88 to 1.00). Notably, *ENO1* and *CAMK2A* shared a high-scoring motif (GGACGTGC), suggesting coordinated regulation, while *TEK*’s unique motif (CGGCGTGT) indicates distinct vascular regulation. These predicted binding sites are consistent with their potential involvement in the HIF-1 signaling pathway, as suggested by functional enrichment analysis. For instance, the circDUSP16–ssc-miR-671-5p–*CAMK2A* axis was predicted as a candidate regulatory relationship, in which circDUSP16 may interact with ssc-miR-671-5p and may be associated with *CAMK2A* expression changes related to calcium signaling under hypoxic conditions. *CAMK2A* is critical for synaptic plasticity, yet its overactivation can exacerbate cellular stress in hypoxic condition*s* [36]. The negative correlation between circDUSP16 and ssc-miR-671-5p (r = −0.83) and the positive correlation between circDUSP16 and *CAMK2A* (r = 0.76) were consistent with the predicted ceRNA relationship. Given the known involvement of calcium signaling in cardiac excitation–contraction coupling and hypoxia-related cardiac responses [37,38], this candidate axis may be relevant to calcium-associated cardiac regulation under hypoxic conditions. However, further experimental validation is required before its regulatory or functional role can be confirmed. Similarly, the circTLK1–ssc-miR-331-3p–*SERPINE1* axis was prioritized as a candidate regulatory relationship, in which circTLK1 may interact with ssc-miR-331-3p and may be associated with *SERPINE1* expression changes related to fibrinolysis and vascular remodeling. *SERPINE1* stabilizes the extracellular matrix and supports vascular integrity in hypoxic environments, a process often upregulated by HIF-1α activation [39,40]. Substantiated by a high positive correlation (r = 0.91), this axis appears redirected toward stabilizing blood vessels and enhancing endothelial resilience in the Tibetan pig heart, providing a novel perspective compared to its broader roles in inflammation or fibrosis [41,42]. Furthermore, the circTLK1–novel-miR-624–*ENO1* axis was identified as a candidate regulatory relationship, in which circTLK1 may interact with novel-miR-624 and may be associated with *ENO1* expression changes related to glycolysis. *ENO1*, encoding alpha-enolase, catalyzes the conversion of 2-phosphoglycerate to phosphoenolpyruvate in glycolysis, playing a central role in energy metabolism and hypoxia tolerance [43,44]. Notably, this axis exhibited the highest degree of synergistic coupling (r = 0.94) in our correlation analysis, indicating a tightly coordinated regulatory response for metabolic reprogramming. This mechanism is consistent with observations in Tibetan pigs’ lung tissues, where miRNAs (e.g., ssc-miR-210) enhance metabolic pathways like glycolysis [45]. Recent research on miRNA expression in Tibetan pig alveolar cells further supports this, demonstrating that miRNAs play a critical role in regulating metabolic pathways under hypoxia, underscoring the significance of the ceRNA network in coordinating metabolic adaptations [46]. The enrichment of *ENO1* and *PFKM* in glycolysis/gluconeogenesis, carbon metabolism, and RNA degradation pathways suggests a dual role in metabolic reprogramming and RNA stability, a phenomenon less explored in prior studies. These findings suggest a potential association between the identified candidate axes and energy metabolism or hypoxia-responsive transcript regulation in Tibetan pigs, consistent with previous genomic studies reporting altitude-associated metabolic features in Tibetan pigs [47]. Further functional validation is required to test this possibility. Additionally, the association of *PFKM* and *CAMK2A* with the glucagon signaling pathway indicates an interplay between energy metabolism and calcium signaling, which may help maintain cardiac function under hypoxic stress. This interplay is particularly relevant in high-altitude adaptation, where energy demands and cardiac stress responses are heightened [48,49].

While our study provides valuable insights, we acknowledge several limitations. The ceRNA network was primarily predicted computationally and requires further experimental validation, such as luciferase reporter assays or circRNA knockdown/overexpression. These targeted functional experiments will be important in subsequent research to further test the proposed molecular interactions. Additionally, focusing on heart tissue offers a targeted perspective but leaves room to explore systemic hypoxic adaptation by examining other tissues, such as the lung, where distinct circRNA profiles have been observed [46]. Future integrative analyses across diverse oxygen-sensitive tissues will be essential to fully elucidate the systemic regulatory coordination of circRNAs. We acknowledge that the limited sample size, with three biological replicates per group, is an important limitation of this study. Therefore, the observed circRNA expression patterns and predicted ceRNA relationships should be interpreted as exploratory and candidate findings. Future studies with larger cohorts are required to confirm these results [50]. This would provide a more comprehensive reflection of the complex genetic architecture underlying high-altitude adaptation. Moreover, comparative analyses with other high-altitude species, such as yaks and Tibetan sheep, could uncover conserved mechanisms of hypoxic adaptation [51,52]. Functional experiments, including knockdown or overexpression of circDUSP16 and circTLK1, could further clarify the mechanistic roles, potentially identifying therapeutic targets for hypoxia-related cardiac conditions [53,54].

## 4. Materials and Methods

### 4.1. Experimental Animals and Sample Collection

The study examined Tibetan pigs and Yorkshire pigs raised at contrasting altitudes, including high-altitude (3000 m; Linzhi, China) and low-altitude regions (100 m; Beijing, China). Tibetan pigs at high and low altitudes were designated as TH and TL, respectively, while Yorkshire pigs at high and low altitudes were designated YH and YL, respectively. The immigrant groups (YH and TL) were derived from populations that had been transferred to the corresponding environments approximately three years prior and had been bred for one generation. From each group, three biological replicates were collected. The selected pigs were unrelated, non-littermate, six-month-old individuals with similar body weights (approximately 60–65 kg) and healthy growth status. Following humane slaughter, heart tissues were immediately collected, snap-frozen in liquid nitrogen, and stored at −80 °C until RNA extraction.

### 4.2. RNA Extraction, Library Construction, and Sequencing

Total RNA was extracted from heart tissue samples using the RNA Pure Tissue Kit (Tiangen Biotech Co. Ltd., Beijing, China) following the manufacturer’s instructions. RNA purity and concentration were assessed using a NanoDrop ND-2000 spectrophotometer (NanoDrop Products, Wilmington, DE, USA). To eliminate genomic DNA contamination, RNA samples were treated with DNase I (Tiangen Biotech Co. Ltd., Beijing, China). Ribosomal RNA (rRNA) was depleted using the Ribo-Zero rRNA Removal Kit (Epicenter, Madison, WI, USA). RNA library construction and sequencing were performed as described previously [24]. Briefly, sequencing libraries were prepared using the NEBNext^®^ Ultra™ Directional RNA Library Prep Kit for Illumina (New England Biolabs, Ipswich, MA, USA) and sequenced on an Illumina NovaSeq 6000 platform (Illumina, San Diego, CA, USA) to generate 150-bp paired-end reads.

### 4.3. Identification and Differential Expression Analysis of circRNAs

Sequencing data quality control was performed using custom Perl scripts. Clean reads were obtained by filtering out adapter sequences, poly-N reads, and low-quality reads. Data quality metrics, including Q20, Q30 scores, GC content, and sequence duplication levels were systematically evaluated. High-quality reads were then aligned to the *Sus scrofa* 11.1 reference genome using HISAT2 (v2.1.0) with default parameters [55]. Unmapped reads were subsequently processed using find_circ and CIRI2 (v2.0.6) for circRNA identification [56]. CircRNA expression was quantified by counting back-spliced junction reads and normalized as SRPBM to account for sequencing depth and transcript length [57]. Differential expression analysis was conducted using DESeq2 (v1.30.1) with negative binomial generalized linear models [58]. Differentially expressed circRNAs (DE-circRNAs) were defined as those meeting the thresholds of |log_2_(fold change)| ≥ 1 and adjusted *p* < 0.05 across all pairwise comparisons of the TH, TL, YH, and YL groups.

### 4.4. Functional Enrichment Analysis of circRNA Host Genes

Functional enrichment analysis of DE-circRNA host genes was conducted using KOBAS software (v3.0) [59]. Kyoto Encyclopedia of Genes and Genomes (KEGG) pathway analysis was performed to identify significantly enriched biological pathways [60], with the *Sus scrofa* reference genome (v11.1) as the background. Statistical significance was determined using Fisher’s exact test with a threshold of *p* < 0.05. Results were visualized using the integrated tools of KOBAS.

### 4.5. Prediction of circRNA-miRNA-mRNA Interactions and ceRNA Network Construction

Interactions among DE-circRNAs, DE-miRNAs, and DE-mRNAs were predicted using miRanda (v3.3a) [61] and TargetScan (v7.2) [62] software, following established computational protocols. DE-miRNAs and DE-mRNAs were integrated from our previously published transcriptomic datasets of Tibetan pig heart tissues [24]. To ensure biological stringency and minimize false-positive interactions, a multi-step filtering strategy was implemented to define hypoxia-adaptive candidate molecules. First, circRNAs were prioritized based on their differential expression patterns across both breed-specific (TH vs. YH) and altitude-specific (TH vs. TL) comparisons. Second, candidate circRNAs were retained only if their corresponding host genes exhibited significant enrichment (*p* < 0.05) in hypoxia-related KEGG pathways. Candidate interaction pairs were further filtered based on expression correlation coefficients (*p* < 0.05). Specifically, we required a negative correlation between circRNA-miRNA and miRNA-mRNA pairs, alongside a positive correlation for circRNA-mRNA pairs, consistent with the ceRNA hypothesis. The final regulatory networks were constructed and visualized using Cytoscape (v3.8.2) [63].

### 4.6. JASPAR Analysis of HIF-1α Binding Sites

To investigate the regulatory role of HIF-1α in the ceRNA network, promoter sequences (2000 bp upstream of transcription start sites) of DE-mRNAs (*ENO1*, *PFKM*, *TEK*, *SERPINE1*, *CAMK2A*) were retrieved from the pig reference genome. Binding motifs were predicted using the JASPAR 2022 core vertebrate database (https://jaspar.genereg.net/ accessed on 7 April 2026) with a relative profile score threshold of 0.80.

### 4.7. circRNA Validation and Quantitative Real-Time PCR (qRT-PCR)

Total RNA was reverse-transcribed into cDNA using the FastKing RT Kit with gDNase (Tiangen Biotech, Beijing, China) according to the manufacturer’s instructions. Divergent primers were designed based on the identified back-splice junction sequences to amplify circRNA-specific back-splice junctions, using cDNA and pig genomic DNA (gDNA) as templates. PCR products were separated by agarose gel electrophoresis and confirmed by Sanger sequencing [64]. The absence of amplification from gDNA was used to support the circular structure of the validated circRNAs. For expression validation, qRT-PCR was performed using a BioRad CFX96 Real-Time PCR system (BioRad, Hercules, CA, USA) with a 20 μL reaction volume containing 10.8 μL of 2× Universal SYBR Green Fast qPCR Mix (Tiangen Biotech Co. Ltd., Beijing, China), 0.4 μL (10 μM) of each forward and reverse primer, and 1 μL of cDNA. The amplification program included an initial denaturation at 95 °C for 15 min, followed by 40 cycles of denaturation at 95 °C for 10 s, annealing at 60 °C for 20 s, and extension at 72 °C for 30 s. HPRT served as the internal control, and relative gene expression was calculated using the 2^−ΔΔCt^ method [65]. Primers were designed using NCBI Primer-BLAST web tool (available at https://www.ncbi.nlm.nih.gov/tools/primer-blast/ accessed on 7 April 2026), which uses Primer3 (v2.5.0) for primer design, and synthesized by SinoGenoMax (Beijing, China) (Table S10).

### 4.8. Data Analysis

All data are presented as mean ± SEM. For qRT-PCR expression data, differences between two groups were analyzed using a paired Student’s *t*-test, while comparisons across the four groups (TH, TL, YH, YL) were performed using one-way analysis of variance (ANOVA) followed by Tukey’s post hoc test. Statistical analyses were conducted using SPSS (version 25.0; IBM, Chicago, IL, USA). Different letters (a, b) indicate significant differences (*p* < 0.05), while the same letter denotes no significant difference. Graphs were generated using GraphPad Prism 8 (GraphPad Software, San Diego, CA, USA).

## 5. Conclusions

In summary, this study identified statistically significant circRNA expression differences in heart tissues of Tibetan and Yorkshire pigs raised under high- and low-altitude conditions. Integrative ceRNA analysis prioritized three candidate circRNA–miRNA–mRNA axes, including circDUSP16–ssc-miR-671-5p–*CAMK2A*, circTLK1–ssc-miR-331-3p–*SERPINE1*, and circTLK1–novel-miR-624–*ENO1*. These axes were associated with HIF-1 signaling, glycolysis, vascular remodeling, and calcium signaling, suggesting their potential involvement in cardiac responses to high-altitude hypoxia. However, given the limited sample size and the computational nature of ceRNA prediction, these regulatory relationships should be considered candidate mechanisms and require further validation using larger cohorts and direct functional assays.

## Data Availability

All data supporting the findings of this study are presented in the article and its Supplementary Files. The raw RNA-seq data are available in the NCBI Sequence Read Archive (SRA) under accession number PRJNA880668.

## Supplementary Materials

The following supporting information can be downloaded at https://www.mdpi.com/article/10.3390/ijms27104392/s1.

## Abbreviations

The following abbreviations are used in this manuscript:

ncRNAs	non-coding RNAs
ceRNA	competitive endogenous RNA
DE lncRNAs	Differentially Expressed lncRNAs
DE mRNAs	Differentially Expressed mRNAs
DE miRNAs	Differentially expressed miRNAs
TH	Tibetan pig living at High altitude
YH	Yorkshire pig living at High altitude
TL	Tibetan pig living at Low altitude
YL	Yorkshire pig living at Low altitude
KEGG	Kyoto Encyclopedia of Genes and Genomes
PCA	Principal Component Analysis
SRPBM	Spliced Reads Per Billion Mapped reads
